# Optimizing the Diagnostic Assessment of Left Ventricular Noncompaction Cardiomyopathy: The Clinical Value of Cardiac Magnetic Resonance Imaging

**DOI:** 10.2174/0115734056440144251119065016

**Published:** 2025-11-25

**Authors:** Xiaogang Xue, Xiaoyong Xu, Xueyao Lin, Gaoyan Wang, Haibo Dong

**Affiliations:** 1 Department of Radiology, Ningbo Medical Center LiHuiLi Hospital , Ningbo, China

**Keywords:** Cardiomyopathy, Magnetic resonance imaging, Feature tracking, Left ventricular function, Left ventricle, Ventricular myocardium, Stroke volume

## Abstract

**Introduction:**

The current diagnostic criteria for noncompaction of the ventricular myocardium (NVM) remain inconsistent, and comprehensive cardiac magnetic resonance (CMR) imaging data on the disease are limited. Therefore, the purpose of this study is to evaluate the clinical utility of CMR imaging in the diagnosis and functional assessment of patients with NVM.

**Materials and Methods:**

Twenty patients with NVM and twenty age- and sex-matched healthy controls (HC) underwent comprehensive CMR imaging. Postprocessing software was used to quantify left ventricular longitudinal strain, both global longitudinal strain (GLS) and strain in the basal, middle, and apical segments (BLS, MLS, and ALS, respectively). Statistical analyses were performed to assess group differences.

**Results:**

Compared with the HC group, patients with NVM presented significantly increased left ventricular end-diastolic volume (LVEDV), end-systolic volume (LVESV), stroke volume (LVSV), and myocardial mass index (LVMI) and a significantly reduced left ventricular ejection fraction (LVEF) (all *P* < 0.001). All NVM patients presented prominent trabeculations and deep intertrabecular recesses in the left ventricle during diastole. Cine imaging revealed direct blood flow communication between the recesses and the ventricular cavity. The myocardium exhibited a thin compacted outer layer (C) and a thickened noncompacted inner layer (NC), with an average NC/C ratio of 2.8 ± 0.5. For these patients, NVM primarily involved the apical and adjacent mid-ventricular free wall segments; in five patients, it also involved the basal segment. Right ventricular noncompaction was observed in five patients, and apical ventricular aneurysms were identified in two patients. Compared with the HC group, the NVM group presented a significantly lower ALS (*P* < 0.05); however, the BLS, MLS, and GLS values were not significantly different between the groups (*P* > 0.05).

**Discussion:**

Our study demonstrated the feasibility of using CMR imaging to quantitatively assess left ventricular systolic function in NVM patients. The choice of longitudinal strain as a primary parameter was driven by the fact that NVM predominantly affects the endocardial myocardium, particularly the subendocardial fibers, which are primarily longitudinal. As such, longitudinal strain is particularly sensitive for detecting myocardial contractile dysfunction in NVM. Our results indicated that ALS apical longitudinal strain is a more significant marker of contractile dysfunction in NVM than MLS, which was not significantly altered in NVM patients relative to HCs.

**Conclusion:**

CMR imaging offers robust diagnostic capabilities for patients with NVM and, when combined with feature tracking, allows the quantitative assessment of left ventricular systolic function. The ALS may serve as a sensitive marker of early myocardial dysfunction and may be clinically important in guiding timely diagnosis and intervention.

## 1 INTRODUCTION

Noncompaction of the ventricular myocardium (NVM), also known as left ventricular noncompaction cardiomyopathy (LVNC), is a rare congenital disorder caused by the arrested myocardial compaction during embryogenesis. It is characterized by prominent trabeculations and deep intertrabecular recesses that communicate directly with the ventricular cavity [1, 2]. Although NVM predominantly affects the left ventricle, biventricular involvement can occur in some patients [3, 4]. Clinically, NVM is associated with heart failure, arrhythmias, and thromboembolic events and is often associated with poor patient outcomes [5, 6]. Despite advances in relevant imaging modalities, the current diagnostic criteria for NVM remain inconsistent, and comprehensive CMR imaging data on the disease are limited. This study aims to evaluate the diagnostic utility of CMR imaging in patients with NVM and to improve understanding of the disease by analyzing clinical and imaging features from a cohort of 20 patients.

## 2 MATERIALS AND METHODS

### 2.1 Clinical Data

A total of 20 patients who were diagnosed with NVM at Ningbo Medical Center LiHuiLi Hospital between June 2019 and December 2024 were retrospectively enrolled. The cohort included 10 males and 10 females, with an age range of 22 to 76 years and a mean age of 41.45 ± 15.87 years. All patients met the echocardiographic diagnostic criteria for NVM proposed by Jenni *et al*. [7], which include (1) a two-layered myocardial structure consisting of a thin, compacted outer layer (C) and a thick, noncompacted inner layer (NC), with excessive trabeculations and deep intertrabecular recesses; (2) a NC/C ratio ≥ 2 at end-systole; (3) direct blood flow from the ventricular cavity into the recesses on color Doppler imaging; and (4) the absence of other congenital cardiac anomalies. Patients with hypertension, coronary artery disease, obesity, diabetes mellitus, or arrhythmias were excluded.

An age- and sex-matched healthy control (HC) group of 20 individuals, including 10 males and 10 females aged 15 to 68 years (mean age 35.75 ± 13.66 years), was also recruited. None of the control subjects had a history of congenital heart disease, hypertension, coronary artery disease, or other structural cardiac disorders.

### 2.2 Sample Size Estimation

Given the rarity of NVM, sample size determination was based on previous literature and traditional estimation methods rather than formal power calculation. The present study aimed to include as many eligible patients as possible within the study period to ensure sufficient statistical validity.

### 2.3 CMR Examination Protocol

CMR imaging was performed with a 3.0 Tesla GE Discovery MR750 scanner (GE Healthcare, USA). All scans employed retrospective electrocardiographic (ECG) gating and were acquired during an end-expiratory breath hold. A series of cine fast imaging employing steady-state acquisition (FIESTA) images was acquired to evaluate left ventricular function. Imaging views included a short-axis view from the base to the apex as well as two-chamber, four-chamber, and left ventricular outflow tract views.

The key scan parameters were as follows: slice thickness of 8 mm, interslice gap of 2 mm, repetition time (TR) of 3.5 ms, echo time (TE) of 1.5 ms, flip angle (FA) of 45°, field of view (FOV) of 360 × 280 mm, image matrix of 216 × 256, and temporal resolution of 40 ms.

Prior to the CMR examination, all patients underwent heart rate and blood pressure assessment. For patients with tachycardia, propranolol was administered as needed to control heart rate, ensuring that it remained below 75 beats per minute and that blood pressure remained below 140/90 mmHg. The acquired images were subsequently screened, and cases with significant arrhythmia that substantially compromised image quality during the CMR examination were excluded from the analysis.

### 2.4 Imaging Postprocessing

Postprocessing was performed using uAI Discover–CMR (Research Version, United Imaging Intelligence Co., Ltd., Shanghai, China), an advanced cardiac imaging analysis program. Endocardial and epicardial contours of the left ventricle were delineated to automatically calculate the left ventricular end-diastolic volume (LVEDV), end-systolic volume (LVESV), stroke volume (LVSV), ejection fraction (LVEF), and myocardial mass index (LVMI). The left ventricular volume curve was plotted based on all cardiac phase maps acquired from the cine sequences, with end-diastole and end-systole defined as the phases corresponding to the maximum and minimum volumes, respectively.

CMR tissue tracking operates by tracking and measuring the displacement of myocardial tissue. On CMR cine sequences, the endocardial and epicardial borders are manually traced, with each border defined as a series of points. By tracking the positions of these points, the myocardial motion trajectory is captured. Through layered algorithmic processing, parameters, such as myocardial tissue velocity, displacement, and strain, are quantitatively derived, enabling the evaluation of myocardial systolic and diastolic function from a mechanical perspective [8].

The pathogenesis of LVNC is characterized by arrested myocardial compaction during embryonic development. It is generally accepted that myocardial compaction occurs sequentially from the base to the apex [9, 10]; therefore, the study focuses on the strain values of each region of the left ventricle.

**Fig. (1):**
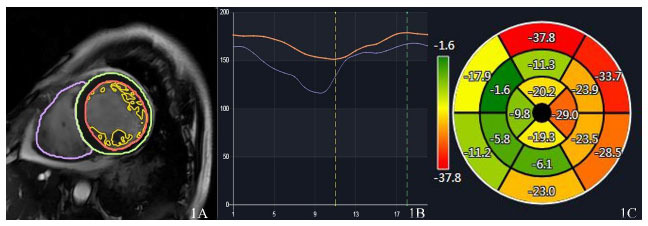
Myocardial strain mapping and annotation. (**A**): Delineation of the left ventricular endocardium, epicardium, and noncompacted myocardium (Left ventricular end-diastole); (**B**): Left ventricular volume curve; (Fig. **1C**): Bull's-eye plot of left ventricular myocardial strain.

The following strain parameters were derived: basal longitudinal strain (BLS), middle longitudinal strain (MLS), apical longitudinal strain (ALS), and global longitudinal strain (GLS) (Fig. **1**).

To ensure measurement reliability, all analyses were performed according to a standardized protocol by two independent cardiologists blinded to clinical data. Inter- and intra-observer agreement was assessed by repeating measurements in 10 randomly selected cases, and mean values were used for final analysis.

### 2.5 Statistical Analysis

All the statistical analyses were performed using SPSS software version 25.0 (IBM Corp., Armonk, NY, USA). Continuous variables following a normal distribution are reported as means ± standard deviations, and group comparisons were performed using one-way analysis of variance (ANOVA). Nonnormally distributed data are presented as medians (interquartile ranges), and group performance was measured using the nonparametric Mann–Whitney U test or the Kruskal–Wallis test, as appropriate. Categorical variables are expressed as counts, and group comparisons were performed with the chi-square test. A two-tailed P value < 0.05 was considered to indicate statistical significance.

## 3 RESULTS

### 3.1 Comparison of Clinical Characteristics between the Noncompaction of the Ventricular Myocardium (NVM) and Healthy Control (HC) Group

A total of 20 patients with NVM were included in the study. Ten NVM patients were male, 10 were female, and the mean age of the group was 41.45 ± 15.87 years. The HC group also included 20 individuals (10 males and 10 females), and the mean age was 35.75 ± 13.66 years. There were no statistically significant differences in age or sex distribution between the two groups (*P* > 0.05) (Table **1**).

Compared with the HC group, the NVM group presented significantly greater LVEDV, LVESV, and LVSV, a significantly reduced LVEF, and a significantly elevated LVMM (*P* < 0.001 for all). These findings indicated that NVM is associated with increased ventricular volume, myocardial hypertrophy, and impaired systolic function.

### 3.2 Imaging Characteristics of NVM Patients

**Fig. (2):**
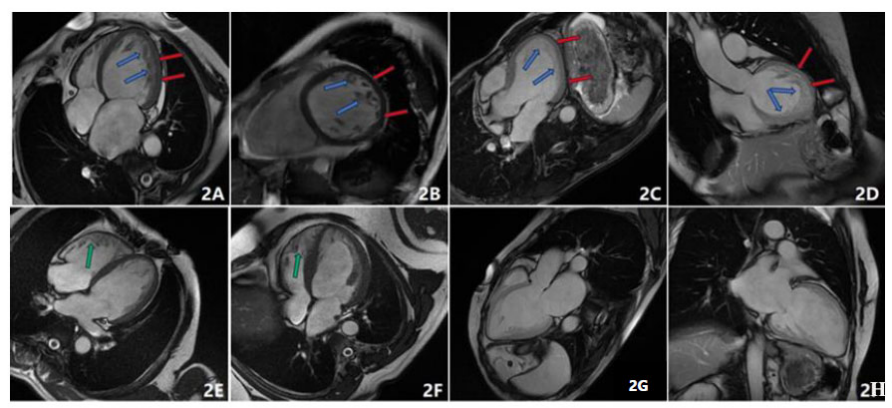
CMR imaging features of noncompaction of the ventricular myocardium (NVM) (Left ventricular end-diastole). (**A**-**D**) Multiplanar CMR images from patients with NVM. The red arrows indicate the thin compacted myocardial layer, whereas the blue arrows denote the markedly thickened noncompacted myocardial layer, which has a reticular or mesh-like appearance with prominent trabeculations and deep intertrabecular recesses; (**E**,**F**) Green arrows indicate involvement of the right ventricle in affected patients; (**G**,**H**) NVM associated with apical ventricular aneurysm, as indicated.

All 20 NVM patients demonstrated the hallmark imaging features of the disease, including multiple prominent trabeculations and deep intertrabecular recesses within the left ventricle during diastole. Cine images revealed that blood flow within the recesses was in continuity with the ventricular cavity (Fig. **2**).

The affected myocardium exhibited a distinct two-layered structure: a thin, compacted outer layer (C) and a markedly thickened, noncompacted inner layer (NC), the latter of which appeared sparse, mesh-like, or reticular in morphology. The mean noncompacted-to-compacted myocardial thickness ratio (NC/C) during diastole was approximately 2.8 ± 0.5.

The lesions predominantly involved the apical and adjacent mid-lateral segments of the left ventricle, whereas 5 patients presented basal involvement. Additionally, 5 patients presented right ventricular noncompaction, and 2 presented apical ventricular aneurysms.

### 3.3 Comparison of Longitudinal Strain Parameters between the Noncompaction of the Ventricular Myocardium (NVM) and the Healthy Control (HC) Group

There were no significant differences in the BLS, MLS, or GLS values between the NVM and HC groups (*P* > 0.05). However, the ALS value was significantly different between the groups (*P* < 0.05), indicating a notable reduction in apical longitudinal contractile function in NVM patients (Table **2**).

## 4 DISCUSSION

### 4.1 Regarding NVM and Diagnostic Methods

NVM is a rare congenital cardiac disorder characterized by impaired myocardial development. The most widely accepted hypothesis regarding its pathogenesis is that the condition arises from a disruption in the compaction of myocardial fibers and trabecular structures between the fifth and eighth weeks of embryonic development [10]. In the later stages, NVM often leads to progressive heart failure, in which cardiac contractile function serves as a critical prognostic indicator. Accurate assessment of myocardial function in NVM patients is essential for predicting patient outcomes and guiding clinical management [11].

Currently, echocardiography is the preferred method for diagnosing NVM and evaluating myocardial function, although it is limited by operator dependency and poor reproducibility, affecting diagnostic accuracy [12, 13]. CMR imaging, with its high soft-tissue resolution, multiparametric capabilities, and excellent reproducibility, serves as a comprehensive, noninvasive approach for evaluating cardiac morphology, function, and tissue characteristics [14].

### 4.2 The Regions Most Commonly Involved in NVM Patients

In this study, the regions most commonly involved in NVM were the left ventricular apex and adjacent mid-wall, whereas the interventricular septum and base were rarely involved, aligning with findings from other studies [15-17]. This pattern mirrors the sequence of myocardial compaction during embryonic development, proceeding from the epicardium to the endocardium and from the base to the apex. Thus, areas that experience compaction later in development are more prone to disruption, resulting in the generation of NVM [9, 10].

### 4.3 Definition of NC/C Ratio Threshold in NVM Diagnosis

There is no consensus on the diagnostic threshold for the NC/C ratio in NVM. Some researchers have suggested that an NC/C ratio≥2 is a reliable criterion; however, this may lead to overdiagnosis, particularly when distinguishing NVM from the excessive trabeculation observed in dilated cardiomyopathy [17, 18]. Other investigators have proposed that an NC/C ratio ≥2.5 may yield a more accurate diagnosis, though this may also lead to missed diagnoses [19]. In this study, the average NC/C ratio measured during diastole was 2.8 ± 0.5.

Due to the unique characteristics of cardiac tissue, achieving an absolute pathological gold standard for the diagnosis of NVM remains challenging, and significant controversy persists regarding the diagnostic NC/C ratio. Although our study proposes a novel diagnostic criterion, the definitive diagnosis of NVM ultimately requires clinicians to integrate and comprehensively analyze various clinical data and examination findings.

### 4.4 CMR Evaluation of Systolic Function in NVM Patients

Our study demonstrated the feasibility of using CMR imaging to quantitatively assess left ventricular systolic function in NVM patients. The choice of longitudinal strain as a primary parameter was driven by the fact that NVM predominantly affects the endocardial myocardium, particularly the subendocardial fibers, which are primarily longitudinal. As such, longitudinal strain is particularly sensitive in detecting myocardial contractile dysfunction in NVM [20].

Our results indicate that ALS apical longitudinal strain is a more significant marker of contractile dysfunction in NVM than MLS, which was not significantly altered in NVM patients relative to HCs. This finding notably contrasts with those of some previous studies, which could be attributed to the relatively small sample size of our cohort, potentially introducing bias.

## 5 STUDY LIMITATIONS

This study has several limitations. First, it is a single-center study with a relatively small sample size; further validation in larger multicenter cohorts is warranted. Second, myocardial strain measurements in patients with NVM were not performed using the conventional 17-segment model but were instead regionally measured based on the distribution of NVM involvement. Although this method is innovative, it requires further refinement. We will conduct larger-scale measurements based on subsequent multicenter experiments and perform comparative analyses with echocardiography to validate the reliability of this innovative measurement approach. Third, CMR tissue tracking has certain technical limitations in measuring myocardial strain parameters. For instance, the relatively low number of image frames acquired within a single cardiac cycle results in limited temporal resolution. Additionally, vendor variability and a lack of standardization, as well as the significant impact of varying degrees of anterior chest wall deformities on biventricular mechanics, may contribute to inaccuracies in strain measurements [21, 22]. Currently, CMR tissue tracking technology continues to evolve, and future technical improvements, such as compressed sensing and artificial intelligence-assisted analysis, are expected to further enhance its accuracy and clinical utility.

## CONCLUSION

This study not only confirms the diagnostic feasibility of CMR for NVM but also highlights the value of combining CMR with tissue-tracking technology for quantitative evaluation of left ventricular systolic function. These findings have significant implications for clinical patient management, aiding in early diagnosis and guiding treatment strategies for individuals with NVM.

## Figures and Tables

**Table 1:** Clinical characteristics of patients with noncompaction of the ventricular myocardium (NVM) compared with healthy controls (HC).

-	**NVM (n=20)**	**HC (n=20)**	***P***
Age(years)	41,45±15.87	35.75±13.66	0.231
Male(%)	10(50%)	10(50%)	1.000
LVEDV(ml)	201.50±71.22	74.61±14.70	<0.001*
LVESV(ml)	124.25±76.25	29.77±7.10	<0.001*
LVSV(ml)	77.20±30.63	44.56±10.03	<0.001*
LVEF(%)	42.10±19.00	59.60±6.29	<0.001*
LVMI(g/m^2^)	109.40±40.87	44.40±9.99	<0.001*

**Abbreviations:** Left Ventricular End-Diastolic Volume (LVEDV), Left Ventricular End-Systolic Volume (LVESV), Left Ventricular Stroke Volume (LVSV), Left Ventricular Ejection Fraction (LVEF), and Left Ventricular Myocardial Mass Index (LVMI).

**Table 2:** Longitudinal strain parameter comparison between patients with noncompaction of the ventricular myocardium (NVM) and healthy controls (HC).

-	**NVM**	**HC**	***P***
BLS	-9.40±5.21	-10.54±6.25	0.534
MLS	-11.32±5.60	-11.04±9.15	0.908
ALS	-12.11±5.49	-17.11±3.03	0.001*
GLS	-10.89±5.29	-13.62±3.81	0.069

**Abbreviations:** Basal longitudinal strain (BLS), Middle longitudinal strain (MLS), Apical longitudinal strain (ALS), and Global longitudinal strain (GLS).

## Data Availability

The datasets used and/or analysed during the current study are available from the corresponding author [X.X] on reasonable request.

## LIST OF ABBREVIATIONS

NVM: Noncompaction of Ventricular Myocardium
CMR: Cardiac Magnetic Resonance
HC: Healthy Controls
LVEDV: Left Ventricular End-Diastolic Volume
LVESV: Left Ventricular End-Systolic Volume
LVSV: Left Ventricular Stroke Volume
LVEF: Left Ventricular Ejection Fraction
LVMI: Left Ventricular Mass Index
BLS: Basal Longitudinal Strain
MLS: Mid Longitudinal Strain
ALS: Apical Longitudinal Strain
GLS: Global Longitudinal Strain
LVNC: Left Ventricular Noncompaction Cardiomyopathy
FIESTA: Fast Imaging Employing Steady-state Acquisition.

## STANDARDS OF REPORTING

STROBE guidelines were followed.
